# Managing complexities of inflammatory rheumatic diseases as a migrant—a qualitative exploration of health literacy experiences

**DOI:** 10.3389/fpubh.2026.1844770

**Published:** 2026-06-29

**Authors:** Laura Treacy, Lise-Merete Alpers, Sarah Hakim, Maryam Azimi, Heidi A. Zangi

**Affiliations:** 1 Diakonhjemmet Hospital, Health Services Research and Innovation Unit, REMEDY - Center for Treatment of Rheumatic and Musculoskeletal Diseases, Oslo, Norway; 2 Centre for Diaconia and Professional Practice, VID Specialized University, Oslo, Norway; 3 Faculty of Health Science, Institute of Nursing and Health, VID Specialized University, Oslo, Norway; 4 Diakonhjemmet Hospital, Patient Advisory Board, Division of Rheumatology and Research, Oslo, Norway

**Keywords:** health literacy, inflammatory rheumatic diseases, language proficiency, migrant health, Ophelia process

## Abstract

**Background:**

Migrants comprise over 17% of Norway’s population and are at increased risk of limited health literacy, a key determinant of health. Health literacy is shaped by language, culture, social norms, discrimination, and health system responsiveness. Inflammatory rheumatic diseases are complex, chronic conditions requiring long-term, specialised care. Patients with these diseases and a migrant background, particularly those with limited proficiency in the dominant language, face additional barriers and poorer outcomes, yet their health literacy experiences remain underexplored. This study explored the experiences of patients with inflammatory rheumatic diseases, a migrant background, and limited language proficiency in accessing, understanding, appraising, and using health information and rheumatology services in Norway.

**Methods:**

Nineteen semi-structured, in-depth interviews were conducted with patients with a migrant background, and who used interpreters during consultations with health professionals. Participants were recruited from rheumatology departments at two Norwegian hospitals. Interviews were audio-recorded, transcribed verbatim and analysed using reflexive thematic analysis. Two patient research partners were involved throughout the study.

**Results:**

Three main themes were generated. First, barriers to accessing and using health information and services were shaped by the interplay between language proficiency, digital literacy, and social support, which often compensated for system gaps. Health systems implicitly assumed a minimum level of linguistic and digital competence, creating mismatches between system demands and patients’ abilities. Second, trust was foundational, shaping engagement and acceptance of treatment. Trust developed over time; length of residency did not necessarily equate to confidence in using services, and trust in health professionals often preceded trust in the wider system. Third, employment was an important factor shaping health literacy and disease management: while colleagues supported service navigation, physically demanding and precariat working conditions constrained access to care and self-management.

**Conclusion:**

Managing these conditions among migrants with limited language proficiency is shaped by interrelated individual, relational, and structural factors. Health literacy is co-constructed through social networks, trust, and contexts such as employment, while system assumptions about language and digital competence create fragmented access. Interventions should move beyond communication barriers to foster trust, support shared understanding, and address broader social and structural conditions influencing engagement with health services.

## 1 Introduction

The number of migrants has nearly doubled since 1990, with estimates exceeding 304 million worldwide (1). People migrate for various reasons, including work, study, family, or they are forced to flee conflicts, persecution or disasters (2). In line with the International Organization for Migration definition, this study refers to migrants as individuals who have moved away from their usual residence, across an international border, temporarily or permanently, and for a variety of reasons (3). In Norway, where this study is set, the number of migrants has more than tripled in the last 20 years (4, 5). Over 17% of the population are migrants, representing more than 200 different countries and territories (6). This study focuses on first generation migrants, using the term, “patients with a migrant background”.

Migrants are at increased risk of limited health literacy (7–11). Health literacy is a multi-dimensional and evolving concept that encompasses competencies related to accessing, understanding, appraising and using health information and services (12). It has been recognised as a determinant of health and a factor related to health outcomes and health promotion (7–11). The WHO conceptual framework for health literacy development highlights that health literacy is shaped by intersecting factors, such as language, norms, cultural and religious beliefs, as well as experiences of racism and discrimination (13, 14).

Contemporary health literacy research has shifted from solely focusing on individual competencies to acknowledging the role and responsibility that health professionals, services, and organisations have in responding to the health literacy needs of their community (7). This includes health literacy responsiveness, referring to the extent to which health professionals and services provide information and care in ways that promote equitable access and support informed decision-making, and organisational health literacy, which looks at whether systems and organisations have the infrastructure, leadership and resources to meet diverse health literacy needs (13, 15). In addition, the concept of distributed health literacy highlights how individuals draw on knowledge and support of family and social networks, framing health literacy as a shared resource rather than an individual skill (16).

The healthcare system in Norway is publicly funded and mainly financed through taxes, with small co-payments for consultations, diagnostic tests, and certain treatments. Once an annual cost ceiling is reached, services become free for the rest of the year (17). Healthcare is semi-decentralised with municipalities being responsible for primary health care, and the state for specialised health care. General practitioners (GPs) act as gatekeepers to secondary services (18). Inflammatory rheumatic diseases (IRDs) are chronic conditions which, if left untreated, lead to irreversible joint damage, impaired functional ability and high risk of comorbidities (19–22). In Norway, patients with IRDs are treated by rheumatologists who work either within hospitals or as private specialists with public contracts. Patients with IRDs will predominantly see a rheumatologist and a rheumatology nurse and some may be referred to a physiotherapist, occupational therapist or social worker if needed (23). Managing IRDs is complex, requiring long-term engagement with both pharmacological and non-pharmacological treatment (24–26). Effective management of IRDs therefore depends heavily on patients’ ability to engage with health information and navigate healthcare services, making health literacy particularly important in this context (27–29).

The growing number and diversity of migrants in Norway has also led to an increasing number of patients with migrant backgrounds within rheumatology care. For these patients, managing IRDs may be further complicated by cultural, linguistic, and systemic barriers. From an intersectionality perspective, these barriers interact with other social identities and positions such as gender, socioeconomic status, legal status, ethnicity, language proficiency, and experiences of social exclusion and stigma, contributing to inequities in access to care and health outcomes (30, 31). Research indicates that migrants, especially those with limited proficiency in the country’s dominant language, are at increased risk of poorer health outcomes (32–34). Within rheumatology specifically, patients with a migrant background have been shown to experience worse prognoses (35–39).

Despite growing research on migrant health, patients with a migrant background and limited proficiency in the country’s dominant language remain underrepresented in rheumatology research. In particular, there is limited knowledge about their health literacy experiences in the context of living with and managing IRDs. Such knowledge is essential for developing equitable and health literacy-responsive rheumatology services. Therefore, the aim of this study was to explore the experiences of patients with inflammatory rheumatic diseases, a migrant background and limited Norwegian language proficiency in accessing, understanding, appraising and using health information and services.

## 2 Materials and methods

### 2.1 Study design

This qualitative study is part of a larger project designed to develop health literacy-informed interventions for patients with IRDs, a migrant background and limited Norwegian language proficiency. The overarching goal of the project is to improve health outcomes in patients with IRDs and a migrant background. The project is inspired by the Ophelia (Optimising Health Literacy and Access) process, a structured, participatory approach that supports the collaborative development of interventions aimed at improving health literacy. Central to the Ophelia process is the active involvement of the target population, ensuring that the interventions reflect their diverse needs, contexts and lived experiences (40). A qualitative study of health literacy typologies is presented in a previous publication (41). This current paper describes findings from the first phase of the Ophelia process.

### 2.2 Study setting, participants and recruitment

Participants were recruited from two rheumatology hospital departments in South-Eastern Norway between December 2023 and October 2024, using convenience sampling techniques. The inclusion criteria comprised patients with a migrant background aged 18 and above, diagnosed with an IRD by a rheumatologist, who used interpreters when communicating with health professionals. This strategy served as a proxy for identifying patients with limited Norwegian language proficiency. We included language groups that had most frequently used professional interpreters at the two departments in the year prior to the study: Arabic, Polish, Somali, Tamil and Urdu. At that time, people from Poland represented the largest migrant group in Norway, and three of the other languages corresponded to some of the largest migrant groups (42). Thus, the selection was informed both by the departments’ patient population and by the demographic relevance of these language groups in the Norwegian context. Forty-two patients who met the inclusion criteria received a leaflet from their rheumatologist or rheumatology nurse in both Norwegian and their mother tongue, inviting them to a face-to-face semi-structured interview. The first author (LT), together with a professional interpreter, spoke with potential participants interested in being interviewed, providing more information about the project, its purpose, and the interview structure.

The study was reviewed as health service research by the Regional Medical Ethics Committee and approved by the hospital’s Data Protection Ombudsman for Research. Participants received information about the project and interviews in both Norwegian and their mother tongue. Participation was voluntary, participants signed an informed consent form and were assured they could withdraw at any time without consequences.

### 2.3 Data generation

A semi-structured interview guide was developed inspired by the Ophelia processes’ Health Literacy questionnaire (40) and Conversational Health Literacy and Assessment Tool (43), in collaboration with rheumatology health professionals and two patient research partners (SH & MA). The interview guide contained questions about participants’ experiences in living with and managing their disease, navigating the healthcare system, communicating with health professionals, finding and understanding health information and services, and social support (see Supplementary file 1).

The individual in-depth interviews were conducted by the first author (LT) alongside professional interpreters. The interviews lasted between 80 and 225 min, with a mean length of 127 min. They were conducted at a time and place most convenient for each participant, such as libraries, cafes, the hospital, and participant’s homes. Reflective notes were written after each interview, detailing the interview process, contextual information, themes discussed and new insights highlighted. These notes were discussed with all the authors and allowed for adjustments to the interview guide throughout the data generation period. For example, questions around work and mental well-being were included after these discussions. Alongside the concept of information power, these analyses helped guide the decision to stop data collection. Assessment of information power was based upon the study aims, quality of the dialogue, specificity of the participants and richness, depth and quality of data (44). Based on these considerations, we determined that data adequacy was achieved after 19 interviews (44). Questions about each participant’s characteristics, such as age, education and years in Norway, were asked at the end of each interview.

### 2.4 Reflexivity and positionality

A key consideration throughout the research process was reflection on how conducting interviews with participants from different linguistic and cultural backgrounds could pose challenges, creating distance and misunderstandings between participants and the interviewer. The interviewer LT’s role as a researcher might have widened this distance. To minimise the perception of LT as holding power, the participants’ roles as experts during the interviews were emphasised. LT’s previous experience working with migrants in vulnerable positions and being a migrant herself provided valuable perspectives during both the interviews and the analysis.

The research team included two Patient Research Partners with lived experiences of managing IRDs as patients with migrant backgrounds, as well as researchers experienced in rheumatology and migration. All five authors were cognisant of their positionality and how previous experiences, interests and opinions would shape their analysis of data and interpretation of findings. Preconceptions were discussed openly, and any disagreements were resolved through collaborative discussion until consensus was achieved. Ongoing reflections on positionality, combined with regular discussions among the authors regarding impressions, emerging interpretations and potential biases, contributed to a rigorous and reflexive analytical process.

An additional methodological reflection concerned the use of interpreters, as interviews conducted with interpreters may also create distance and lead to potential misunderstandings. Interpreting from one language to another will inevitably involve some interpretation of what is said, as languages have their own idiosyncrasies and nuances that cannot always be translated from one language to another (45–47). Therefore, interpreters risk influencing the message or meaning of a conversation. To address this, we undertook several strategies to ensure that the meaning in the participant’s message was conveyed as directly as possible. We used only professional interpreters with experience in healthcare settings. LT met with the interpreters before each interview to discuss the study’s overall aim and the purpose of the interviews. The role of the interpreter in in-depth research interviews, as opposed to consultations with health professionals, was also discussed. The interview guide was reviewed and discussed with each interpreter, and the aim of the more open questions, designed to elicit narrative responses when appropriate, was highlighted. Ongoing dialogue after each interview and during the analysis process allowed for the discussion and resolution of uncertainties. When re-listening to the audio recordings, any uncertainties that arose were discussed with all authors, and when necessary, the main author listened again to the audio recordings with the relevant interpreter in order to clarify the translation and meaning of the participant’s response. When uncertainties remained, LT and the interpreter contacted the relevant participant to revisit and clarify them. In total, two participants were contacted to revisit or explore in greater depth topics where uncertainties had arisen or new themes that had emerged in later interviews.

### 2.5 Data analysis

Data were transcribed verbatim in Norwegian by LT and analysed abductively according to Braun and Clarke’s reflexive thematic analysis (48). The analysis was an iterative process, involving movement between six different phases, as codes were reviewed and re-reviewed, and subsequent themes and sub-themes were altered. 1. Data familiarisation: LT and the last author (HAZ) read all transcripts and reflective notes, the second author (LMA) read a sample of the transcripts. New reflective memos were written and discussed with all authors, highlighting key thoughts and impressions from the entire dataset, as well as potential initial themes. 2. Coding: NVivo 14 software was used to organise the data. LT systematically coded the dataset line by line, capturing both explicit and implicit meanings within the data. 3. Generating initial themes: this was a reflective and collaborative process that identified shared and recurring meanings across the dataset, with initial themes generated based upon the data, as well as previous insights, knowledge of the literature and research field. 4. Developing and reviewing themes: the potential themes were reviewed in relation to the coded extracts, as well as the dataset as a whole, ensuring coherence and clear distinctions between themes. These potential themes were reviewed and modified during discussions with all the authors. 5. Refining, defining and naming themes: The themes were further adapted, developed and refined. These refinements occurred through discussions with all the authors, as well as being presented and discussed at two separate research group meetings with researchers experienced in qualitative methods, rheumatology and/or migrant research. 6. Writing up: Themes and subthemes were presented along with relevant or descriptive quotes from the data. All quotes were translated from Norwegian to English by LT, who is fluent in both languages. These translations were quality-assured by two of the other authors (HAZ & LMA), who are also fluent in both languages.

## 3 Findings

The 19 participants had received their IRD diagnosis between 4 months and 30 years ago and had resided in Norway for 7 to 37 years. All but one lived full-time in Norway and all but one were diagnosed in Norway. Participants described experiences from both recent and earlier periods of living with an IRD. Table 1 shows participants’ characteristics.

**Table 1:** Characteristics of participants.

Characteristic	Category	N
Gender	Female	10
	Male	9
Age in years	31–40	1
	41–50	10
	51–60	6
	61 +	2
Language	Arabic	4
	Polish	5
	Somali	4
	Tamil	2
	Urdu	4
Diagnosis	Rheumatoid arthritis	13
	Psoriatic arthritis	1
	Spondyloarthritis	2
	Gout	2
	Polyarthritis	1
Length of time with diagnosis	<1 year	2
	1–10 years	12
	11–20 years	0
	21 + years	5
Education	Primary education or less	4
	Secondary education	9
	Vocational or higher education	6
Employment status	Employed	5
	Studying	1
	Unemployment benefits	7
	Disability benefits/pension	6
Years in Norway	≤ 10	6
	11–20	4
	21 +	9

The data analysis generated three main themes and sub-themes (Table 2).

**Table 2:** Themes and sub-themes.

Themes	Sub-themes
Theme 1: The interplay of barriers and facilitators to accessing, understanding, and using healthcare	1.1 Language, digital and social factors in accessing and using healthcare 1.2 Challenges in understanding health information and disease management
Theme 2: Trust as a foundation for care	2.1 Learning to trust a new system and build trusting relationships 2.2 Undermining trust
Theme 3: The connection between employment and health literacy	3.1 Work as a facilitator for accessing health information and navigating the healthcare system 3.2 Work as both beneficial and challenging for health

### 3.1 Theme 1—the interplay of barriers and facilitators to accessing, understanding and using healthcare

#### 3.1.1 Language, digital and social factors in accessing and using healthcare

Participants highlighted how limited Norwegian language proficiency, limited digital literacy and weak social networks interacted to shape their ability to access and use health information and services. It was rarely one factor alone, but the combination of them that determined how easily they could navigate the system.

Limited language proficiency affected participants’ ability to express needs, understand rights, and identify and use available services. One participant described the cumulative challenges of living in a new country without language or system knowledge:

*When you are new in the country and don't know the language, there are many challenges you face, and the biggest one is the language, when you can't express yourself enough to tell the doctor how you feel. And if you have an appointment somewhere, you don't know how to get there, how the system works, how the buses work, and what to do. So, you have many challenges that, in a way, make things go much slower than they should do. (P14)*

Many participants tried to overcome the challenges created by limited language proficiency by using digital translation tools to translate written health information and navigate the online health system. This required a baseline level of digital competence that not all participants had. Therefore, for some participants the combination of limited Norwegian and limited digital literacy meant they struggled to independently book appointments, use HelseNorge—the official online portal and digital gateway to the Norwegian healthcare system—or read letters, Short Message Services (sms) or information from healthcare providers.

In a highly digitalised society such as Norway’s, limited digital skills not only meant challenges translating health information but also using everyday services such as online banking, navigating government and welfare services, as well as using public transport. Those who had family members who were more skilled both digitally and in the Norwegian language often relied on them for help. For example, as one participant explained when discussing HelseNorge:

*“What is HelseNorge? My children probably know. I do not” (P18).*

These examples illustrate how the Norwegian healthcare system tacitly assumes a minimum level of Norwegian proficiency and digital literacy. When these assumptions were not met, significant gaps emerged between what the system expected of patients and what they were actually able to do, leading to challenges in independently navigating and using services.

Family members often played an important role in bridging these gaps. Participants with relatives who were digitally competent and proficient in Norwegian could ‘borrow’ both digital and linguistic capital. Family members helped them manage their appointments, interpret letters or messages, and improve communication with health professionals. One participant described why he preferred to attend rheumatology appointments with his wife:

*It is also an advantage to have my wife with me at meetings and appointments. Because I have a poor memory. So, I bring my wife, who remembers, and she has a better understanding of the language and speaks better Norwegian and that makes it easier for me (P8)*

Another participant emphasised how her daughter could convey her situation more effectively:

*My daughter can explain in a good way what I have been through and the challenges I face (P19).*

These experiences can be understood as examples of ‘distributed health literacy’, where the capacity to access and use health information and services is located within family networks rather than in the individual alone. While this social support mitigates some barriers, it may also create dependency and potential vulnerability when such support is absent.

The use of interpreters represented another important strategy for addressing language barriers. In Norway, patients have the right to a free, qualified interpreter if they cannot communicate adequately in Norwegian. However, participants reported variations in how this right was realised in practice. Some had been unaware of their right to interpreter services. Others were aware and proactively requested one, while some explained that health professionals decided whether they needed an interpreter or not. Some participants expressed frustration or shame about needing an interpreter due to limited Norwegian proficiency. Others believed their Norwegian proficiency was sufficient and preferred not to have everything interpreted during appointments to avoid wasting time. Several recalled situations in which interpreters were not used, resulting in poor communication or misunderstandings.

Although most participants were satisfied with the professional interpreter services, some held back certain nuances and details when communicating through an interpreter. Some proactively sought health professionals who spoke their language or felt reassured knowing that a health secretary at the clinic could speak their language. Others chose to bring a family member to hospital appointments who could assist with communication, as well as provide advocacy and emotional support.

Interpreting services are available during scheduled consultations between health professionals and patients, but they are not readily accessible when patients contact the hospital from home. This creates a fragmented pattern of access to health services. For example, one participant expressed reluctance to contact the hospital for advice or assistance, despite having the rheumatology department’s direct number.

*What also demotivates me a bit about using the telephone services, is that I understand even less on the phone than I would in person (P10)*

For participants without a close social network with strong Norwegian skills, these gaps in support could contribute to delayed help-seeking.

Taken together, these experiences suggest that patients with limited Norwegian proficiency, digital literacy and weak social networks face multiple, intersecting challenges. The Norwegian healthcare system tacitly assumes a minimum level of Norwegian proficiency and digital literacy. When these assumptions are not met, responsibility shifts to patients and their families to bridge these gaps. For those without a strong social network this exacerbates inequities to access to and use of healthcare services. This highlights the importance of health professionals exploring patient’s language, digital literacy and social support, but also underlines the need to address system-level barriers, ensuring that services are accessible without advanced language or digital skills or relying on family members.

#### 3.1.2 Challenges in understanding health information and disease management

Some participants noted that while they could read written health information in Norwegian, they often struggled to comprehend its content, illustrating how basic literacy does not guarantee adequate health literacy skills. One participant shared her experience:

*I was illiterate (…) but now I can read and write. I can read Norwegian, for example, what is written on that paper there, but I don’t understand the meaning. So, if I receive letters, I usually look at them and read them, but I do not understand what is written. (P14)*

Other participants highlighted that translating or interpreting information into their dominant language was not always sufficient; they still faced challenges in understanding the information provided. This suggests a need for support to translate information into a meaningful understanding of their condition, including biomedical concepts and the practical implications of disease management.

Some participants expressed uncertainty about their diagnosis, often referring to it simply as “rheumatism.” Others used terms associated with IRDs, such as “inflammation,” but were unsure what they meant. This may indicate a mismatch between participants’ lay explanatory models of illness and biomedical terminology, complicating shared understanding with health professionals. One participant, who had lived with her disease for over 20 years, described her last appointment with her rheumatologist:

*The doctor said that there is a lot of wear and tear and a little inflammation (…) I do not even know what that means (P16).*

This persistent lack of understanding, even among participants with over 20 years of disease experience suggests that long-term contact with specialist services does not necessarily translate into improved conceptual understanding of IRDs or into shared understanding between health professionals and patients.

Some participants struggled not only with medical terminology but also with understanding their disease and its long-term management. They expressed uncertainty about how the disease affected their bodies “on the inside*”* and about how medications were intended to control disease activity and alleviate symptoms. In particular, several participants appeared to interpret treatment as a time-limited cure rather than ongoing management of a chronic illness, shaping their expectations of what medications should achieve. One participant expressed her confusion regarding prescribed medication:

*I was told that Methotrexate stops the inflammation. I thought, okay, I will continue taking Methotrexate for a certain period and then the inflammation will be over. But then I find out that the inflammation is spreading, (…) "So why did you say the first time that it stops the inflammation?" (P12)*

Here, the expectation that Methotrexate would “stop” the inflammation contrasts with the biomedical understanding of IRDs as chronic conditions that may fluctuate over time, requiring continuous and adaptive treatment. Another participant described how side effects and limited knowledge of the consequences of not taking her medication had led her to prioritise traveling to warmer climates over medical care. She subsequently blamed herself for her disease “spreading” because she had not adhered to her treatment.

*Some of the blame may also be mine, because I perhaps didn't have a good enough understanding of the illness at the beginning. I was given medication, but those medications had side effects, such as stomach pain, so I didn't take them. Then the illness spread. If I had the same knowledge I have now at the start, I would have taken my medications from the beginning. Earlier, I thought, "Okay, if I am just out in the sun enough, maybe it will help to get a lot of sun” (P11)*

This account shows how participants drew on prior understandings of illness and treatment (e.g., warmth and sun as therapeutic) when biomedical explanations felt incomplete or when side effects were too difficult to manage. It also illustrates a tendency towards self-blame for disease progression, which may obscure the role of limited, non-tailored information and support from the healthcare system.

These findings illustrate how differing perceptions of illness and treatment can lead to misunderstandings between health professionals and patients. Across participants, such misunderstandings did not only involve specific medical terms but also the chronic nature of IRDs, the goals and duration of treatment, and the consequences of non-adherence. In this situation, it resulted in the participant missing appointments and discontinuing medication over several years.

This theme demonstrates how barriers to accessing and using health information and services are not experienced in isolation but emerge through the interplay between language proficiency, digital literacy, and access to social support. While interpreters are vital for enabling communication, they do not by themselves ensure adequate comprehension or a shared understanding between health professionals and patients. Furthermore, this theme suggests that current communication practices and explanations of the disease and its management may be insufficient to ensure the level of shared understanding that is needed for effective long-term disease management.

### 3.2 Theme 2—trust as a foundation for care

#### 3.2.1 Learning to trust a new system and build trusting relationships

All participants had grown up with healthcare systems that differed from the Norwegian system. Many were unfamiliar with the roles of multidisciplinary team members who typically work within Norwegian rheumatology services, such as occupational therapists and social workers, and did not know how to access these services.

For some, receiving a diagnosis of an IRD represented their first experience navigating the Norwegian healthcare system. This is a vulnerable time to be learning how to navigate complex services, which can lead to increased dependency on health professionals or social networks. One participant explained that despite living and working in Norway for over 12 years, she had never needed to use the healthcare services until she received her rheumatic diagnosis. She noted it took time to learn how to use the system and to trust it and the people working within it. She explained that her trust in her rheumatologist developed before she fully understood how the system functioned:

*I gained a lot of trust in her when I still didn’t understand the Norwegian system and how the specialists work here in Norway (P9)*

This quote suggests that trust develops sequentially, first at the interpersonal level, and later, at the institutional level. Participants described several factors contributing to trusting relationships with their health professionals: taking time for their patients, being available and welcoming when needed, being open to questions and concerns, addressing those concerns seriously, offering concrete advice for managing symptoms and medication side effects, and demonstrating empathy. Furthermore, some participants viewed relational trust as a two-way process requiring health professionals to learn to trust their patients as well, with continuity of care being important. As stated clearly by one participant:

*I can summarise that we should have a permanent rheumatologist (P13)*

Many preferred face-to-face communication over video consultations, reflecting a need for relational contact as a foundation for trust, particularly among migrants who are less familiar with the system. This raises challenges in health systems where there is an increasing use of digital and remote consultations.

Once these trusting relationships were established, participants felt confident their health professionals would provide reliable information about their disease and relevant services. For some, trust meant that even if they did not fully understand how their medication worked or had concerns about long-term side effects, they chose to take their medication based on their confidence in their doctor. One participant explained that he believed his health professional offered more reliable guidance than other sources of information:

*I trust the doctor more, and I believe that the doctor can better inform me about any potential side effects I can expect, what risks are involved in taking these medications, than trusting the package insert from the manufacturer. I trust the doctors here (at the hospital) 100 % and I know that if they say something, then that is how it is (P7).*

Participants also trusted their health professionals to make the best treatment decisions. One participant expressed reliance on his doctor to ensure he was prescribed the most suitable medication for his condition:

*"They know the system and the hospital and the healthcare providers (…) they know that if new methods or new medications come out, they know what works for me and what they can do for me. So, I am putting all the responsibility on them."* (P15)

This quote indicates that, for some participants, trust led them to hand over full responsibility to their health professionals for information needs and decision-making regarding their care. This may reduce decisional burden on the patient or feelings of being overwhelmed, but it may also mask the need for more information and limit opportunities for shared decision-making and informed choice, foundational concepts in patient-centred care.

#### 3.2.2 Undermining trust

Unmet expectations and poor communication undermined participants’ trust in the healthcare system and in individual health professionals. One participant described how his expectations for appointments with his rheumatologist were not met, expressing frustration that his treatment regime remained unchanged from one follow-up appointment to the next:

*I have met him (the doctor) up to 10 times, and he has only said, "you have the medicine, just use it like that” and then I just go* (P4)

After perceiving no change in treatment or acknowledgement of his concerns, he began to regularly miss follow-up appointments, as he did not see the value in attending them. This suggests that disengagement from care may be a consequence of mistrust rather than indifference to one’s health. A few participants recounted situations where they felt their GP did not take them seriously, leading to delayed diagnoses. One participant linked the delay in diagnosis and subsequent treatment directly to her limited language proficiency:

*I feel that when a person doesn't speak the language, when you go to the GP and are unwell, they don't take you in right away. It takes a long time. You have to come back many times before you get help. When you become very sick, that's when you're taken seriously and referred on. (…) It's very important to convey that those who have language challenges really struggle to get treatment promptly (…). I know several people who go to the same doctor and get the same response every time, even though they are sick they are not taken seriously. (P14)*

This quote underscores how some participants felt they were not taken seriously by health professionals, perhaps due to their limited language proficiency and status as a migrant, which impacted timely care and undermined their trust in the Norwegian healthcare system. For some participants, having a family member accompany them to an appointment and act as an advocate resulted in their GP taking their concerns seriously. Delayed response to symptoms and repeated dismissal, particularly among those with limited language proficiency and without close social networks to advocate for them, may be perceived as a form of structural exclusion rather than isolated episodes of poor communication. This perceived structural exclusion may reinforce feelings of marginalisation and impact future use of healthcare services.

This theme underscores how trust is foundational for effective care, influencing patients’ willingness to accept treatments, maintain relationships with health professionals, and engage with the healthcare system. Length of residency in a country does not necessarily equate to familiarity with or confidence in using health services. Trust takes time to build and usually develops sequentially, with trust in individual health professionals typically preceding trust in the wider system. Therefore, the role of health professionals and their ability to build rapport and foster a trusting relationship may be even more important in the context of treating patients with a migrant background and limited language proficiency.

### 3.3 Theme 3—the connection between employment and health literacy

#### 3.3.1 Work as a facilitator for accessing health information and navigating the healthcare system

Several participants explained that participation in working life helped them gain knowledge about the Norwegian healthcare system, indicating that health literacy is shaped not only within healthcare encounters but also through everyday social relationships at work. One participant explained that his boss had helped him access health services when he first started experiencing symptoms of his IRD:

*(…) it was my boss who arranged it, because at that time I didn't have a clue about things. I don't speak any English, and I hadn't started learning Norwegian (…) (P10)*

Another participant recounted how a colleague advised her to request a referral to a specific rheumatology department. The same colleague informed her about the availability of rehabilitation in warmer climates for people with certain chronic diseases:

*My colleague told me (about the rehabilitation), the rheumatologist didn't mention it, nor did the doctor. I heard it from a lady in the office who said she received this rehabilitation, so I could also apply (P13)*

These experiences suggest how informal interactions with colleagues facilitate access to services and information not always provided by health professionals.

#### 3.3.2 Work as both beneficial and challenging for health

Many participants described being employed as directly beneficial for their health, contributing to physical activity and positively impacting their mental well-being. Conversely, unemployment could negatively affect mental well-being, with participants describing feelings of low mood or depression, and adversely affecting finances. Experiences of financial strain, in turn, made it harder for some patients to manage their disease, as they could not afford physiotherapy sessions or gym memberships. One participant, unable to work and relying on unemployment benefits, described how his limited finances affected his ability to attend physiotherapy, and he did not have the confidence to train without professional supervision:

*I used to go to a physiotherapist for quite a while. At the New Year, I couldn't continue, and I said: “Sorry, I don’t have the finances to come to you in the same way as before.” I have a limited income, and the benefits I have are just enough for rent, expenses, and living costs. (P3)*

This indicates how economic vulnerability acted as an active barrier shaping participants’ ability to enact recommended health behaviours. A loss of employment can result in a cycle in which reduced income limits access to supportive care, potentially worsening disease outcomes. In turn, worsening symptoms may further restrict work capacity, reinforcing a cyclical process of ill health and socioeconomic disadvantage.

Those who had been working prior to developing their IRD, typically had physically demanding jobs, such as working as cleaners or in the construction industry. Some were no longer able to remain in these jobs, while others who were still working described how their work either contributed to increased pain or limited their ability to manage their condition. For example, one participant explained that although she had been advised to exercise, after a full day’s work, she did not have the energy:

*I work as a cleaner (…) so it’s a bit tough, and when I get back home, I must sleep because I’m so tired. My problems aren’t that I have a lot of pain, but that I’m fatigued all the time. (…) If I sleep for 11 hours, yes, I get a little energy, but if I sleep for 8 hours, I have no energy (P13)*

This quote highlights the challenges in following clinical advice about self-management activities, such as exercise, when combined with the realities of having a physically demanding job and experiencing fatigue. Employment sometimes prevented participants from accessing services and opportunities to better manage their condition. For example, patient education courses and exercise sessions were typically offered during working hours. One participant explained how a rheumatology nurse had helped her find a physiotherapist under the National Insurance scheme, relieving her of the full costs. However, the physiotherapist’s appointment times conflicted with her work hours, leading to complaints from her boss about her absences. She was unable to find another physiotherapist with the same financial agreement outside work hours, and ultimately, she discontinued treatment. This indicates how the structural organisation of services—such as daytime-only appointments—can make adherence to recommended care difficult for those in less flexible jobs, inadvertently penalising those who remain in employment.

A few participants believed that the focus on finding or maintaining employment delayed their referral to a rheumatologist and, subsequently, their IRD diagnosis. One recalled his appointment with his GP:

*When I told my doctor about my symptoms, she was, in a way, under pressure from the Norwegian Labour and Welfare Administration (NAV) who had said: “We have sent a person to you, so that you can examine him, medicate him, and send him back to work as soon as possible.” I told the doctor that it is difficult for me to go to work because I have swelling in both my hands and feet, but she said, “No”, she had received a message from NAV saying that “you must send him back to work” (P17)*

This suggests how appropriate specialist referral may be delayed because of system-level pressures to maintain participation in paid work.

This theme highlights how work shapes both health literacy and disease management. Interactions with colleagues can positively influence patients’ ability to access and use health information and services, which is particularly important for migrant patients with limited experience within the Norwegian healthcare system. Yet employment can be a double-edged sword: supporting mental well-being, social ties, and access to and use of health information and services, while also increasing physical strain, limiting access to care, and sometimes delaying diagnosis. Working conditions, including the type and flexibility of work, affected participants’ ability to follow advice and use services to help manage their disease.

## 4 Discussion

This study aimed to explore the experiences of patients with inflammatory rheumatic diseases, a migrant background and limited language proficiency in accessing, understanding, appraising and using health information and services. The analysis generated three themes: (1) the interplay of barriers and facilitators to accessing, understanding and using healthcare (2) trust as a foundation for care and, (3) the connection between employment and health literacy. Together these themes illustrate how managing an IRD with a migrant background can be complex. Limited Norwegian language proficiency intersects with trust and employment, resulting in distinctive and, at times, challenging health literacy experiences.

### 4.1 Overcoming language differences does not guarantee a shared understanding

IRDs are complex, and challenges can arise for all patients in understanding the disease and its management (24–26). Our study found that these challenges are exacerbated for migrants with limited proficiency in the dominant language, particularly when unfamiliar concepts or medical terminology are used. Languages possess varied colloquial and dialectic expressions that can influence how people interpret and understand concepts and terms (49, 50). Additionally, diverse cultural backgrounds among speakers of the same language mean that correct translation or interpretation of information from one language to another does not ensure linguistic and cultural congruence (45, 51), nor guarantee a shared understanding between patients and health professionals. Without this common understanding, migrants’ ability to actively engage in healthcare interactions is limited, compromising shared decision-making and self-management. The responsibility for ensuring a common understanding lies with health professionals (52). This emphasises the importance of “health literacy responsiveness” of health services in recognising and accommodating diverse health literacy needs, as well as creating environments that enable and support all patients to access and engage with health information and services (7, 14).

### 4.2 Trust and relational aspects of health literacy

Our findings suggest that health literacy among patients with migrant backgrounds is influenced by trust in health professionals and the broader healthcare system. Participants’ willingness to accept and implement advice, despite limited comprehension, often stemmed from their confidence in the professionalism and responsibility of health professionals and the healthcare system. These findings align with Brooks et al., who noted that trust influenced participants’ readiness to accept and implement healthcare messages and attend healthcare services (53). Building trusting relationships was deemed invaluable in meeting participants’ health literacy needs, reinforcing the concept that health literacy is situated within relational contexts (53, 54).

Several elements that foster trusting relationships depend on the health professional’s comportment, communication skills, and ability to establish rapport. Trusting relationships are important for understanding and respecting cultural differences and varying perceptions of health and illness, ultimately facilitating culturally responsive care (50, 55). Our findings emphasise that developing trust and rapport between patients and health professionals from different linguistic and cultural backgrounds often requires more time, especially when interpreters are involved. Continuity in care and in-person communication were frequently preferred, a finding consistent with previous research (50, 53, 54, 56, 57). This poses challenges when services cannot guarantee appointments with the same rheumatologist and when remote monitoring is increasingly utilised in rheumatology healthcare (58). Aligned with other studies, including research with migrants and refugees (53, 59–63), issues such as poor communication, unmet expectations and negative past experiences undermined trust. This highlights the need for health literacy initiatives targeting migrant populations to prioritise building trust, ensuring continuity of care, and exploring patients’ expectations and prior experiences (61, 64).

Ethnic discrimination is a relatively common experience among migrant and ethnic minorities (65–67). However, in our study, only one participant explicitly discussed experiences of discrimination in the form of racism. Although a few participants described experiences that could be interpreted as discriminatory, none of them labelled these situations as such. This may be because discriminatory experiences are often subtle enough that one does not feel confident in saying out loud that it was discrimination (65). Another explanation could be that participants did not feel comfortable discussing this theme, partly because LT, who conducted the interviews, is a white migrant and has therefore been unlikely to have experienced racism or discrimination herself. In addition, as a physiotherapist and health researcher, she represents the Norwegian healthcare system, and participants may not have felt comfortable criticising health professionals or the healthcare system. Since ethnic discrimination can have detrimental effects on health and well-being (63, 67–69), the role of racism and discrimination in relation to trust and health literacy warrants further exploration.

### 4.3 Employment and integration shaping health literacy in migrants

Participation in the labour market and mastering the Norwegian language are considered key elements of integration in Norway (70, 71). Integration and health literacy are intertwined; as noted by Fredriksen and Wångdal “Health literacy can be an important tool to help migrants achieve good health and a good integration process” (72), p. 22. Conversely, successful integration can positively influence health literacy. Our findings extend this understanding by demonstrating how employment can serve as a source of social network, shaping migrants’ ability to find, navigate and engage with health information and services. Health literacy is influenced not only by individual skills and motivation, but also by structural realities around migrants. This aligns with a study from Gele et al., who found that employment was an independent predictor of adequate health literacy among Somali women in Norway (73).

Since work is viewed as fundamental to integration in Norway, securing and maintaining employment is especially important for many migrants, particularly those who have migrated for work or whose residency is dependent on paid employment. Chronic diseases, such as IRDs, can severely impact quality of life and functional ability, thereby affecting participation in paid employment (19–22, 74–76).

Participation in paid work is increasingly recognised as an important outcome in the management of IRDs (31). It underpins economic independence, self-esteem, social participation, and overall quality of life, all of which are tightly connected to disease management and shape patients’ function, mental well-being, and engagement in treatment. Sustaining or resuming employment typically depends on adequate symptom control, management of fatigue, and adjustments to the workplace. While participation in paid work is important for all people with IRDs, migrants in Norway face higher rates of underemployment and unemployment compared to the majority population, making job-seeking and retention even more challenging (5, 77–79).

It is important to note that migrants are a heterogeneous group, with significant differences in employment opportunities. Nonetheless, within the Norwegian context, many migrants have work that is repetitive and physically demanding. They often work alone with limited influence over their working day and are more likely to have temporary positions (80). Furthermore, migrants are generally at risk for lower socioeconomic status, even if employed, as they are more likely to earn what is classified as “low-income” compared to the majority population (79). Research emphasises the role of social determinants in health literacy, with socioeconomic status being a key factor (81, 82). Stormacq et al. (83) explored the health literacy experiences of socioeconomically disadvantaged people in Switzerland, noting that barriers to obtaining health information and quality of doctor-patient communication follow a social gradient. Previous research also indicates that there is a social gradient for health literacy (84), with individuals from lower socioeconomic status backgrounds receiving less information, fewer explanations, less listening time, and less advice than patients from higher social classes (83, 85). Therefore, being a patient with lower socioeconomic status—exacerbated by employment challenges, a chronic health condition and migrant status—can increase the risk of experiencing poorer quality in doctor-patient communication, likely compounded by limited proficiency in the dominant language.

Our findings demonstrate how employment can directly affect people’s ability to apply health literacy skills in daily life. For example, being employed provides colleagues as sources of health information and assistance in accessing services. However, employment may also hinder following advice, such as attending exercise classes or education courses offered only during working hours. This suggests that limited engagement or non-adherence should not be assumed to stem from a lack of interest, motivation or understanding. Instead, it may reflect challenging or precarious working conditions and limited integration and social networks, highlighting the need to also address structural barriers.

### 4.4 Intersection of the themes

Previous research in migrant health suggests that a longer duration of residence in a country is often associated with improved health literacy and a greater ability to navigate the health system (73, 86, 87). Our findings provide a more nuanced picture by showing that many years of residence does not necessarily lead to higher health literacy or engagement with health services. For some participants, long-term residence did not guarantee engagement with all aspects of society, including health services. For others, living with their condition and using Norwegian health services for over two decades, did not always translate into an adequate understanding of their disease, nor did it consistently lead to the use of online health services.

Instead, the consequences of long-term residence were shaped by participants’ social and familial circumstances. For some, having lived in Norway for many years meant that they had close family members—often adult children—who were fluent in Norwegian, familiar with the health system, and digitally literate. These family members helped them navigate services and engage with health professionals. By contrast, participants who had also lived in Norway for a long time but had been out of the workforce for many years due to their disease often described limited social networks. This may have constrained their access to information and support, contributing to inadequate health literacy and affecting how they managed their condition.

These findings challenge the notion that many years of residence in Norway will, in itself, positively and uniformly affect health literacy among patients with a migrant background. Rather, the impact of duration of stay is shaped by the broader social context, including work participation, family resources, and social networks.

Taken together, our findings suggest that migrants’ health literacy is shaped by the intersection of relational experiences and communication within healthcare, and structural conditions outside it. Participants’ ability to engage with health professionals and use health information and services was influenced not only by their capacity to understand and comprehend the information provided but also by the level of trust they had in their health professionals and the wider healthcare system. Feelings of shame associated with limited Norwegian language proficiency may have prevented participants from asking questions or requesting further clarification about health information and services. Additionally, factors such as the type, flexibility and security of their work could affect their ability to prioritise health. Thus, facilitators and barriers such as language and social support, as well as trust and employment, do not function as separate influences but as intersecting factors that determine meaningful participation and engagement in healthcare.

Our findings have implications for how health literacy is conceptualised and addressed in migrant contexts. Health literacy should not be viewed merely as an individual skill but rather as emerging from the intersection of healthcare relationships and broader sociodemographic realities, particularly for those facing multiple challenges. Approaches must ensure that information is tailored to individual needs, foster shared understanding, and recognise and address the broader conditions shaping patients’ ability to engage with and utilise health information and services. These approaches could include, for example: (1) the provision of multilingual visual materials in a variety of formats to meet individual information needs, and (2) the systematic use of the communication tool “teach back” to improve shared understanding and foster trust between health professionals and patients.

### 4.5 Strengths and limitations

The findings from our study should be considered alongside certain limitations. We included only five different language groups, which may limit the transferability of the findings to other patients with a migrant background in rheumatology settings. Additionally, we only recruited participants who used interpreters when communicating with health professionals. We recognise that some people with limited language proficiency may choose not to use interpreters for various reasons, such as a lack of trust in the confidentiality of interpreters or a preference for family members over strangers for interpretation. Thus, our findings may not be transferable to all individuals with limited language proficiency.

However, the diversity of the participants regarding cultural and linguistic backgrounds, ages, educational attainment, types of IRD, and lengths of time living in Norway, along with varied experiences of using interpreters, may allow our findings to resonate with other migrant populations experiencing similar chronic health issues in different contexts. Moreover, including participants from diverse cultural and linguistic backgrounds enabled us to identify commonalities in the experience of “being a migrant”, rather than focusing solely on cultural explanations for the health literacy needs of participants.

Two Patient Research Partners, with lived experiences managing IRDs as patients with migrant backgrounds, have been involved since the project’s conceptualisation. They helped develop the idea for this study and were actively involved in data collection by contributing to the interview guide, developing strategies for recruiting participants, as well as participating in data analysis. Regular discussions among the five authors regarding potential biases, differing interpretations, and impressions ensured thorough and reflective analysis of the data. Collectively, these strengths enhanced the study’s rigour and bolstered the credibility of our findings. Understanding participants’ perspectives and lived experiences through in-depth interviews and reflexive thematic analysis not only provides a nuanced understanding of health literacy in a migrant context but also contributes to the literature on migration issues in health literacy with an understudied population. This understanding will be valuable for developing future health literacy interventions.

## 5 Conclusion

Managing inflammatory rheumatic diseases as a migrant with limited proficiency in the country’s dominant language can be complex. This complexity is influenced by intersecting factors such as language, trust, and work, highlighting that health literacy develops relationally and structurally. Some of these factors are migrant-specific and ought to be understood and considered when developing health literacy-informed interventions. The findings from this study provide a nuanced understanding of health literacy in a migrant context and will inform the development of interventions tailored to this patient group.

## Data Availability

The datasets presented in this article are not readily available because they contain information that could compromise the privacy of research participants. Requests to access the datasets should be directed to lauraclaire.treacy@diakonsyk.no.
