# Supplementary material for: Managing complexities of inflammatory rheumatic diseases as a migrant—a qualitative exploration of health literacy experiences
Source: Front Public Health. 2026 Jun 29;14:1844770. doi: 10.3389/fpubh.2026.1844770 (PMC13357978; doi:10.3389/fpubh.2026.1844770)
Supplement: Supplementary file 1 [file Data_Sheet_1.PDF]

Supplementary Material

1 Supplementary Data

SUPPLEMENTARY FILE 1 – Semi-structured interview guide

Thank you for taking the time to talk with me. We want to learn about your wishes and needs for information, support, and follow-up regarding your condition.

The information, experiences and ideas you share will be used to help improve the services we offer for people with inflammatory rheumatic diseases.

There are no right or wrong answers; you answer as best as you can based on your own experiences. If you need to stop, just let me know at any time.

1. Can you tell me a bit about yourself? (e.g. language – which ones/how many, education, work – Norway/other countries, living situation, family/social network, how long in Norway, interests/hobbies).
2. Can you tell me about how you got your diagnosis? (Prompts: how long had you had symptoms? Who did you speak to? How did you get referred to (name of hospital)?
3. How is it to live with your illness? (Prompts: does it impact different aspects of your life? E.g. relationships, roll in the family/with friends, work, economy? How do you tackle these changes/challenges? Have you needed work to be adapted to your symptoms/needs? If so, can you tell me about this? What brings you joy and/or energy?).
4. Can you tell me about a typical day for you?
5. How do you feel you are coping with your condition? (Prompts: is there anything that makes it difficult for you? Anything that makes it easier? E.g. family, friends, healthcare professionals?).
6. Can you tell me a little about what you do to take care of your health? (Prompts: physical activity, diet, sleep habits, medications and treatments, mental well-being, emotional well-being).
7. Can you tell me about the treatment you receive now? (Prompts: how often and where? Who do you meet? E.g. pharmacist, GP, physiotherapist, occupational therapist, social worker).
8. What happens when you have an appointment with the doctor/nurse/physiotherapist? (Prompts: do you get to talk about what is important for you? Do you feel understood by the healthcare professionals? Are you receiving the help or follow-up that you need? How do you prefer to receive follow-up for your condition?)
9. How do you decide which medicine or treatment is best for you? (prompts: do you feel included in your treatment? If yes, in which way? If no, can you tell me what happens? Do you feel the doctor/healthcare professional has different priorities to you? If yes, how do you tackle this?)

10. Can you remember a situation where it was difficult to talk with a healthcare professional? If yes, can you tell me about it? Can you tell me about a situation where you felt it was easy or good communication with a healthcare professional?
11. Have you been invited to “revmaskole” (a specific course offered at the hospital) or another course to help you learn how to live with your disease? (Prompts: if yes, can you tell me about it? If no, would you be interested in this? Why/why not?)
12. How is it for you to understand the healthcare system here in Norway? (Prompts: how is it the same/different from what you are used to? Is there anything difficult? Do you trust the healthcare system/people who work in it?).
13. Do you use other types of treatment or services? (Prompts: abroad? Training/exercise centres?)
14. How do you contact the rheumatology department if you have questions?
15. How is it for you to use an interpreter? (Prompts: have you ever wanted an interpreter but did not have one? Can you tell me about a time when it was difficult with an interpreter?
16. Besides healthcare professionals, who else do you talk to about your health/condition? (Prompts: who do you talk to if you are unsure if you should go to your GP if you don’t feel well? Do you have someone you can talk to if you are unsure about something e.g. medications?
17. Do you receive support from family or friends? (Prompts: what type of help? Can someone accompany you to your appointments? Is the support useful and/or wanted?)
18. How have you found out about your disease? (Prompts: internet, community, specific groups? How do you want to receive information?)
19. How do you decide what is right or correct information for you? (Prompts: information/people you trust more than others? Someone to discuss with? Have you read/received conflicting advice?)
20. What could help you understand information better and find out what is best for you?

21. Do you have anything else you would like to tell me about today?

(Note, not all of these questions will be asked. The questions can be asked in a different order, depending upon how/what the participant talks about)
